# The protective effect of caffeic acid phenethyl ester in the nephrotoxicity induced by α-cypermethrin

**DOI:** 10.1515/med-2023-0781

**Published:** 2023-08-11

**Authors:** Gokhan Nur, Emrah Caylak, Haci Ahmet Deveci, Pinar Aksu Kılıcle, Ayla Deveci

**Affiliations:** Department of Biomedical Engineering, Faculty of Engineering and Natural Sciences, Iskenderun Technical University, Hatay, Turkey; Department of Biochemistry, Faculty of Medicine, Girne American University, Kyrenia, Cyprus; Nutrition and Dietetics Department, Faculty of Health Sciences, Gaziantep University, Gaziantep, Turkey; Department of Molecular Biology, Faculty of Science and Arts, Kafkas University, Kars, Turkey; Department of Property Protection and Security, Vocational School of Technical Sciences, Kilis 7 Aralık University, Kilis, Turkey

**Keywords:** α-cypermethrin, caffeic acid phenethyl ester, histopathology, weight, oxidant, antioxidant

## Abstract

Alpha cypermethrin (α-CYP) is an insecticide, a member of the group of synthetic pyrethroid pesticides. This study aims to assess the histopathological and biochemical subacute effects of α-CYP on the renal tissues of 48 male Spraque–Dawley adult rats. In this study, the rats were divided into six groups: control, α-CYP (10 mg kg^−1^), α-CYP (20 mg kg^−1^), caffeic acid phenethyl ester (CAPE) (10 µmol kg^−1^), α-CYP + CAPE (10 mg kg^−1^), and α-CYP + CAPE (20 mg kg^−1^) groups. The percentage of weight gain was found to be dose-dependent on α-CYP in all groups. As a result of exposure, the normal histological structure of renal tissue was also observed in the control and CAPE groups, while glomerular atrophy and haemorrhage, enlargement of Bowman capsule, glomerular lobulation, and degeneration in distal and proximal tubules were noted in the α-CYP-treated groups with an increased frequency and severity in parallel with the dose increase. Although the severity and intensity of lesions decreased in the α-CYP + CAPE groups, they were statistically insignificant (*p* > 0.05). A decrease in the antioxidant parameter levels and an increase in oxidant parameters were observed in parallel with the negative effects of the antioxidant system in the α-CYP-treated groups. The groups exposed to CAPE in combination with α-CYP exhibited a therapeutic trend towards normalization in biochemical parameters due to the antioxidant character of CAPE. However, considering the statistical difference between the groups treated with α-CYP alone and CAPE alone, it was observed that the therapeutic features of those chemicals were not robust.

## 1 Introduction

The rapid growth of the world’s population after the industrial revolution has resulted in insufficient agricultural products that are not equally available to people everywhere in the world. Therefore, studies on each factor that may cause harvest loss, especially in agricultural products, are of primary importance for the future of people. Among primary reasons, the use of pesticides that prevent the growth of these crops and developed to fight against harmful microscopic and macroscopic organisms that consume them in the food chain has led to an increase in yields since the 1950s.

Although pesticides are very useful in fighting against agricultural pests when used for their intended purpose, occupational exposure, accumulation in soil, and contamination of groundwater and aquatic ecosystems pose a threat to the health of living organisms and the environment other than the pests they are intended to attack [1,2,3,4]. α-Cypermethrin (α-CYP) belongs to pyrethroids and is widely used as agricultural and residential tools. Although they are highly toxic to insects, their most significant advantages are their low toxicity due to the rapid metabolism in mammals and their inability to accumulate in soil [5,6]. The main mechanism of selective toxicity of pyrethroids is that the sodium channels of the nervous system of insects are highly susceptible to pyrethroids. Symptoms of intoxication and neurophysiological studies suggest that this group affects conduction in axons in the nervous system. It is assumed that they keep the sodium channels in the membranes of nerve cells open and so paralyze and kill insects [7,8]. α-CYP is used against crop pests in agricultural production, as well as ectoparasitic in animals, for the control of fleas and other insects in animal shelters, as well as for the control of pests in public health [9,10,11]. Caffeic acid phenethyl ester (CAPE) is an active polyphenol of propolis. It has many pharmacological activities such as anti-inflammatory, free radical scavenger, anti-apoptotic, anti-carcinogenic, and antioxidant [12,13,14,15].

This study aims to assess the effectiveness of CAPE against the nephrotoxicity that α-CYP, one of the factors that adversely affect environmental health, may induce in organisms other than the target organism.

## 2 Materials and methods

### 2.1 Experimental design

The male Spraque–Dawley rats (220–260 g) used in the study were procured from the Experimental Animal Research Centre of Kafkas University. The rats were kept at an ambient temperature of 21°C, with 12/12 h light/dark cycle, and fed with standard rat feed (containing 21% crude protein) and tap water. The subjects were 48 rats, 8 in each group. In the beginning of the study, the animals were randomly divided into six groups provided that they had similar mean weights. All the animals were weighed both at the beginning and end of the study. The rats were divided into a control group in which corn oil was given as solvent (1 ml), groups in which 10 and 20 mg kg^−1^ α-CYP dissolved in corn oil were gavaged, and a group in which 10 µmol kg^−1^ CAPE was injected intraperitoneally (ip), different doses of α-CYP (10 and 20 mg kg^−1^) and CAPE groups to which they were applied together were formed. In these groups, CAPE was administered intraperitoneally into the abdomen, and α-CYP was administered into the stomach by gavage, respectively. In the groups in which different doses of α-CYP and CAPE were administered together, only CAPE was applied for the first 3 days in order to reach sufficient amounts in the body. Then, different doses of α-CYP and CAPE were applied for 27 days. All of the treatments were continued for 30 days. When the experimental protocol was completed, the rats were anesthetized with ketamine hydrochloride/xylazine (80/10 mg/kg) via the intramuscular route; intra-cardiac blood samples were collected and the rats were sacrificed through cervical dislocation and dissected. Renal tissues were collected for histological analysis.

### 2.2 Biochemical analysis

Blood samples drawn for plasma collection were centrifuged at 3,000 rpm for 10 min and stored at −20°C until analysis. Plasma total antioxidant status (TAS) and total oxidant status (TOS) analyses were measured with a spectrophotometer using commercial kits (REL Assay Diagnostics, Gaziantep, Turkey) through the method developed by Erel [16,17]. Oxidative stress index (OSI) was calculated by dividing TOS values by TAS values. As the TAS result was in mmol/L, it was converted to µmol/L before calculation. The malondialdehyde (MDA) levels were measured according to the method reported by Yoshioka et al. [18], TSA was measured according to the method reported by Sydow [19], and nitric oxide (NO) was measured with a spectrophotometer according to the method reported by Miranda et al. [20].

### 2.3 Histopathological analysis

At the end of the study, kidney tissues of the rats sacrificed by cervical dislocation under anaesthesia were extracted and fixed in 10% formalin solution for 48 h for histopathological analysis. As a result of the routine tissue follow-up procedures, they were embedded in paraffin blocks; 4 µm-thick sections were taken from each block. The preparations for histopathological analysis were stained with haematoxylin–eosin (H&E) and analysed under a light microscope (Zeiss Primo Star). The sections were rated as none (−), mild (+), moderate (++), severe (++++), and very severe (++++) according to the lesions in the histopathological findings [21].

### 2.4 Statistical analysis

SPSS (Statistical Package for Social Sciences 22.0 software was used to assess the findings. The quantitative values were analysed by one-way analysis of variance in the statistical data program. The Tukey HSD multiple comparison test was used to evaluate the statistical significance between the groups. The value of *p* < 0.05 was used as a significance threshold. The results were expressed as mean ± standard deviation.


**Ethical considerations:** This study was approved by the Animal Experiments Local Ethics Committee of Kafkas University (approval no: KAÜ-HADYEK/2022-184).

## 3 Results

### 3.1 Live weight findings

After the rats used in the study adapted to the laboratory setting, their live weights were measured both at the beginning of the experiments and at the end of the fourth week. Table 1 shows the body weight changes in the groups throughout the study. When the live weight profiles were examined, it was observed that there was a weight gain in all of the groups compared to the baseline value based on the baseline and last weight measures. When compared with the control group, the weight gain was observed to be less in the groups treated with α-CYP. The live weight gain and its percentage increased in parallel with the increasing dose of α-CYP. Given the increase of 29.48%, in the control group, it is observed that the live weight gain was at a very low level (17.65%) in the group treated with 20 mg kg^−1^ α-CYP. Compared to live weight gains of 20.89 and 17.65% in the groups treated with 10 and 20 mg kg^−1^ α-CYP, the gains of 19.68 and 15.85% in the groups in which CAPE was injected with both doses of α-CYP indicated that CAPE had no effect on the live weight gain.

**Table 1:** Live weight changes observed in the groups on the first and last days of the experiments

Live weight measures	Groups (*n*:8)					
	Control group (corn oil, 1 ml)	CAPE group (10 µmol kg^−1^)	α-CYP (10 mg kg^−1^)	α-CYP (20 mg kg^−1^)	α-CYP + CAPE (10 mg kg^−1^α-CYP + 10 µmol kg^−1^ CAPE)	α-CYP + CAPE (20 mg kg^−1^ α-CYP + 10 µmol kg^−1^ CAPE)
	Mean ± SD	Mean ± SD	Mean ± SD	Mean ± SD	Mean ± SD	Mean ± SD
Baseline weight (g)	234.57 ± 6.3	226.31 ± 3.7	238.32 ± 7.2	230.27 ± 6.9	243.91 ± 9.05	245.4 ± 7.05
Final weight (g)	303.77 ± 5.1	297.53 ± 4.08	288.13 ± 5.2	270.63 ± 6.0	291.93 ± 8.5	284.35 ± 6.4
Weight gain (g)	68.93	71.23	49.8	40.35	48.02	38.95
Weight gain percentage (%)	29.48	31.46	20.89	17.65	19.68	15.85

*n*: number of subjects in the group, SD: standard deviation.

### 3.2 Histological findings

The examination of the preparations from the kidney tissues at the end of the study suggested that CAPE alone is unable to treat these lesions and is insufficient to protect against the damage caused by different doses of α-CYP in the kidney tissue. Table 2 shows the tissue transformations of the histopathological lesions in the kidney tissue.

**Table 2:** The tissue transformations of the histopathological lesions in the kidney tissue

Lesions of kidney tissue	Groups					
	Control group (corn oil, 1 ml)	CAPE group (10 µmol kg^−1^)	α-CYP (10 mg kg^−1^)	α-CYP (20 mg kg^−1^)	α-CYP + CAPE (10 mg kg^−1^α-CYP + 10 µmol kg^−1^ CAPE)	α-CYP + CAPE (20 mg kg^−1^ α-CYP + 10 µmol kg^−1^ CAPE)
Glomerular atrophy	−	−	++	+++	++	++
Infiltration	−	−	+	+	+	+
Congestion					+	+
Enlargement of Bowman capsule					+	++
Glomerular lobulation	−	−	−	++	++	+
Tubular degeneration	−	−	+	++	+++	++

−: no anomaly, +: low frequency of abnormality, +++: moderate frequency of abnormality, ++++: high frequency of abnormality.

**Figure 1:**
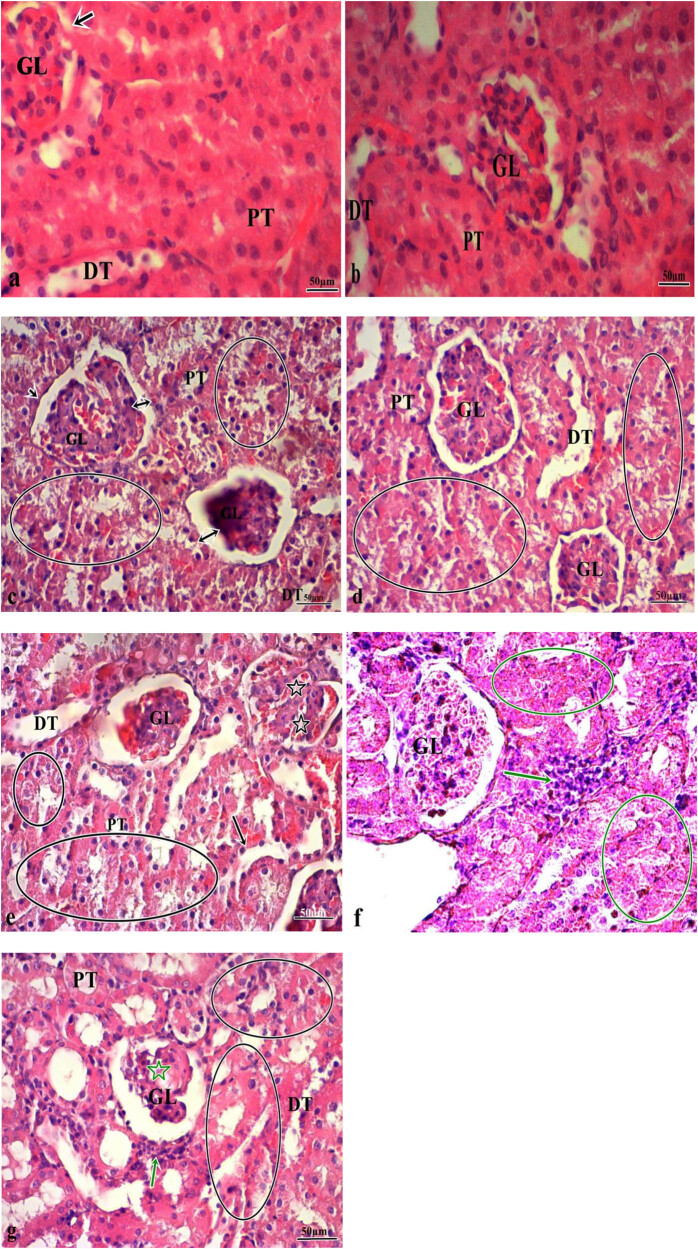
(a and b) Kidney tissue from rats in the control and CAPE groups. Glomerular structure and tubules had a normal appearance (GL: glomeruli, PT: proximal tubule, DT: distal tubule, bowman capsule [arrowhead]). (c) Kidney tissue in 10 mg kg^−1^ α-CYP group (GL: glomeruli, PT: proximal tubule, DT: distal tubule, diffuse tubular degeneration [circle], enlargement in bowman capsule and glomerular atrophy [double-sided arrowhead]). (d) Kidney tissue in 10 mg kg^−1^ α-CYP + 10 µmol kg^−1^ CAPE group (GL: glomeruli, PT: proximal tubule, DT: distal tubule, diffuse tubular degeneration [circle]). (e and f) Kidney tissue in 20 mg kg^−1^ α-CYP group (GL: glomeruli, PT: proximal tubule, DT: distal tubule, diffuse tubular degeneration [circle], separation in the basal lamina [arrows], glomerular lobulation [asterisk], inflammatory cell infiltration [green arrow], diffuse areas of necrosis [green circle]). (g) Kidney tissue in 20 mg kg^−1^ α-CYP + 10 µmol kg^−1^ CAPE group (GL: glomeruli, PT: proximal tubule, DT: distal tubule, diffuse tubular degeneration [circle], glomerular atrophy [green star]). H&E. Bar: 50 µm.

After the routine fixation and tissue follow-up procedures, the kidney tissues were embedded in paraffin blocks. About 5 µm-thick serial sections were sliced from these blocks with a microtome and stained with H&E and then examined under a light microscope. The sections from the groups indicated that the glomerular structure was normal and there was no degeneration in the tubules in the groups treated with solvent (control group) and CAPE (Figure 1a and b). In the groups that were treated with different doses of α-CYP (10 and 20 mg kg^−1^), degeneration in the distal and proximal tubules, enlargement of Bowman capsule, glomerular atrophy, separation of the basal lamina, glomerular lobulation, and inflammatory cell infiltration were increasingly observed in a direct proportion to the increased dose (Figure 1c, e and f). Although there was a relative reduction in lesion severity and frequency in the kidney sections from the groups treated with α-CYP and CAPE (10 mg kg^−1^ α-CYP + 10 µmol kg^−1^ CAPE and 20 mg kg^−1^ α-CYP + 10 µmol kg^−1^ CAPE) compared to the groups treated with α-CYP alone, this was not statistically significant (*p* > 0.05) (Figure 1d and g).

### 3.3 Biochemical findings

**Figure 2:**
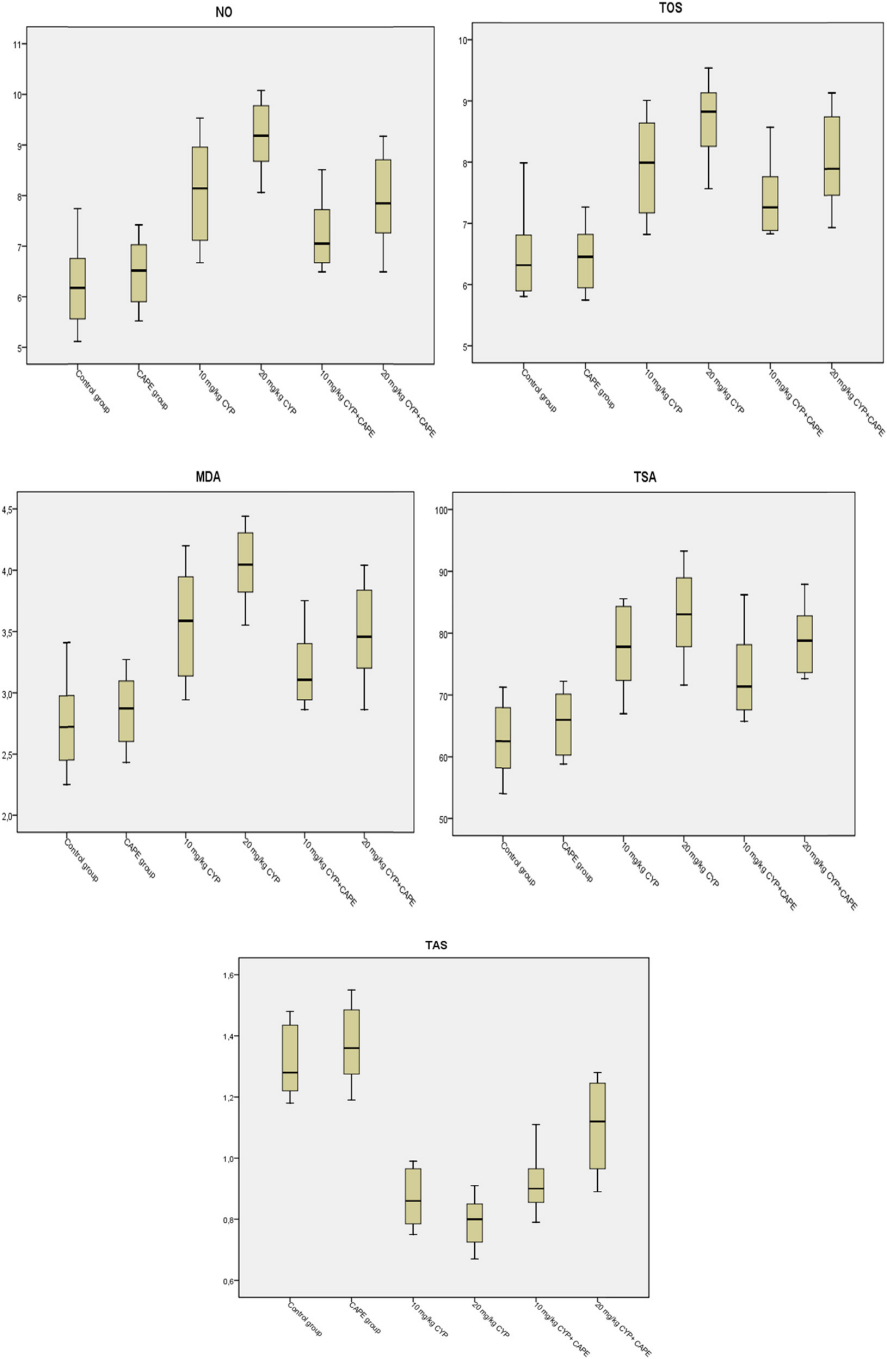
Box plot display of CAPE on TAS, TOS, MDA, TSA, and NO values in rats treated with α-CYP.

When the biochemical data from the study were evaluated, the values in the control and CAPE groups were normal and close to each other. When the groups were analysed for TAS, no difference was observed between the control and CAPE groups (*p* > 0.05). Also, there was no statistical difference between the groups treated with 10 and 20 mg of α-CYP and the 10 mg α-CYP + CAPE group (*p* > 0.05). The statistical difference between the 20 mg α-CYP + CAPE group and all the other groups was significant (*p* < 0.01). Also, the control and CAPE groups were statistically different from all other groups (*p* < 0.01). The difference between the control, CAPE, and 10 mg CYP + CAPE groups for TOS was insignificant (*p* > 0.05). The statistical difference between the 10 mg α-CYP group and 10 mg α-CYP + CAPE, and 20 mg α-CYP + CAPE groups was insignificant (*p* > 0.05). The difference between the group treated with 10 and 20 mg doses of α-CYP and the 20 mg α-CYP + CAPE group was insignificant (*p* > 0.05). The assessment of MDA and NO levels between the groups indicated that they were parallel with those in TOS. The statistical difference between the 20 mg α-CYP group and the 20 mg α-CYP + CAPE group was significant (*p* < 0.01). When TSA levels were analysed, there was no difference between the control and CAPE groups (*p* > 0.05). The difference between the 10 mg α-CYP group and the 10 mg α-CYP + CAPE and 20 mg α-CYP + CAPE groups was insignificant (*p* > 0.05). Also, the statistical difference between the groups treated with α-CYP at single doses (10 and 20 mg α-CYP) and the 20 mg α-CYP + CAPE group was insignificant (*p* > 0.05). No difference was observed between the control and CAPE groups as a result of OSI analysis. Also, the difference between the 10 mg α-CYP group and the 20 mg α-CYP + CAPE group was insignificant (*p* > 0.05) and those two groups were statistically different from all the other groups (*p* < 0.01). The difference between the 20 mg α-CYP group and the other groups and between the 10 mg α-CYP + CAPE group and all the other groups was significant (*p* < 0.01). Table 3 and Figure 2 show the data of the groups.

**Table 3:** The levels of TAS, TOS, MDA, TSA, and NO of the groups and the statistical significance between the groups

Parameters	Groups					
	Control (*n*:8)	CAPE (*n*:8)	10 mg kg^−1^ α-CYP (*n*:8)	20 mg kg^−1^ α-CYP (*n*:8)	10 mg kg^−1^ α-CYP + CAPE (*n*:8)	20 mg kg^−1^ α-CYP + CAPE (*n*:8)
	Mean ± SD	Mean ± SD	Mean ± SD	Mean ± SD	Mean ± SD	Mean ± SD
**TAS (**mmol Trolox equivalent/L)	1.31 ± 0.11^a^	1.37 ± 0.12^a^	0.87 ± 0.09^b^	0.79 ± 0.08^b^	0.91 ± 0.09^b^	1.10 ± 0.15^c^
**TOS** (µmol H_2_O_2_ equiv./L)	6.48 ± 0.75^c^	6.43 ± 0.54^c^	7.93 ± 0.83^a,b^	8.69 ± 0.65^a^	7.40 ± 0.62^b,c^	8.03 ± 0.78^a,b^
**MDA** (μmol/L)	2.74 ± 0.38^c^	2.85 ± 0.29^c^	3.55 ± 0.46^a,b^	4.04 ± 0.30^a^	3.18 ± 0.32^b,c^	3.48 ± 0.40^b^
**TSA** (mg/dL)	62.84 ± 6.07^d^	65.50 ± 5.27^c,d^	77.70 ± 6.97^a,b^	83.07 ± 7.24^a^	73.29 ± 7.35^b,c^	78.88 ± 5.54^a,b^
**NO** (μmol/L)	6.22 ± 0.86^c^	6.47 ± 0.67^c^	8.07 ± 1.05^a,b^	9.17 ± 0.69^a^	7.23 ± 0.72^b,c^	7.91 ± 0.91^b^
**OSI** (Arbitrary Unit) (TOS/TAS × 10)	0.49 ± 0.03^d^	0.47 ± 0.04^d^	0.91 ± 0.06^b^	1.10 ± 0.08^a^	0.81 ± 0.10^c^	0.73 ± 0.04^b^

*p* < 0.01 = statistically significant difference; a, b, c, d: The difference between groups with different letters in the same row is significant; *n*: number of subjects in the group; SD: standard deviation.

## 4 Discussion

α-CYP is an important type II pyrethroid pesticide widely used to protect crops against pests and insect infestations. However, α-CYP toxicity is a risk to both human health and other living organisms in nature [22]. Pyrethroids, to which α-CYP belongs, are sodium channel modulators. They exert their effects on insects through contact and ingestion. Although pyrethroids act as specific insecticides, they are highly toxic to certain nontarget species [23].

The kidney is a complex organ that is involved in many important functions such as the excretion of metabolic wastes, regulation of body water and salt, providing proper acid balance, and secretion of various hormones [24]. Alalwani [22] indicated that CYP was toxic to mammals even at doses of 1/50 and 1/100 of the LD50 of CYP, as it degenerated distal and proximal tubule cells in kidney tissues of rats, and that the toxic character increased in direct proportion to the administered dosage. Another study reported that β-CYP abnormally altered renal histomorphology and ultrastructure, induced renal DNA damage, and triggered renal inflammation [25]. In their study, Grewal et al. [26] administered different doses of CYP (5 and 20 mg kg^−1^) and they observed diarrhoea, low feed intake, body weight loss, and histopathological lesions in organs in the rats. Another study reported a significant loss of body weight due to treatment with 500 mg kg^−1^ CYP [27]. The data of the present study also indicated that α-CYP led to a reduction in the rate of live weight gain. The chemical or radioactive substances that people use or are exposed to lead to the production of free radicals and reactive oxygen species (ROS), suppression of the activities of antioxidant enzymes, and ultimately disruption of the oxidant/antioxidant balance. Taha et al. [28] observed histological symptoms of deformation in kidney tissues in their study on rats treated with 1/50 of the LD50 dose of CYP and reported that it dropped superoxide dismutase (SOD) and glutathione *S*-transferase (GST) levels and significantly elevated MDA and protein carbonyl content in biochemical parameters. Abdou et al. [29] reported that α-CYP caused histological transformations in the liver and kidney. They found an elevation in MDA, lactate dehydrogenase, aspartate aminotransferase (AST), alanine aminotransferase (ALT), urea, and creatinine levels, all of which indicate the presence of oxidative stress, and a reduction in glutathione (GSH), a parameter that indicates antioxidant levels as a result of α-CYP treatment on rats. However, improvements were observed in biochemical parameters and histological appearance in the groups treated with sesame oil + α-CYP depending on the alleviation of oxidative stress. A study on the treatment at different doses of α-CYP (62.5, 125, and 250 mg kg^−1^) reported a reduction in the percentage of body and organ weight gain, a drop in GSH levels, a decrease in mitotic index, and an elevation in the levels of AST, ALT, MDA, blood urea nitrogen, creatinine, micronucleus, and chromosomal aberrations in the groups treated with CYP, in parallel with the increased dose when compared to the control group [30]. Abdul-Hamid et al. [31] reported that 30 mg kg^−1^ α-CYP reduced the activity of antioxidant enzymes SOD and GPx, and elevated the MDA levels of lipid peroxidation markers in albino rats. Eraslan et al. [32] observed an elevation in MDA and NO levels and a drop in SOD, GST, GSH, and CAT levels in rats treated with 80 mg kg^−1^ α-CYP for 12 days. Numerous studies have been conducted that focus on the antioxidant character of CAPE and its protective effect on renal pathologies and nephrotoxicity [33,34,35,36]. In contrast to the elevated CAT and MDA concentrations due to α-CYP treatment, it was reported that CAT and MDA levels dropped in the groups treated with α-CYP + CAPE compared to the group in which only α-CYP was administered [37]. While elevated levels of oxidative markers TOS, MDA, NO, TSA, and OSI were observed in the present study, supporting the findings of other researchers, TAS values dropped as the oxidant/antioxidant balance was disrupted in favour of oxidants.

## 5 Conclusion

The present study was designed to investigate the induction of oxidative stress by α-CYP in the rat kidney. In the groups treated with sub-LD50 doses of α-CYP, which is widely used in agricultural activities, either the production of ROS increased or oxidative stress intensified due to reduced ROS scavenging capacity. Consequently, the levels of oxidative stress markers TOS, TSA, MDA, NO, and OSI elevated and the TAS levels dropped. Therefore, α-CYP is a pyrethroid insecticide that promotes multifaceted toxicity in non-target organisms. α-CYP causes nephrotoxicity, especially as a result of inducing renal DNA damage and renal inflammation. CAPE, which is used as a preservative, is considered to act by affecting the transcriptional and/or translational pathways of antioxidant enzymes in addition to blocking ROS. As a result of these effects, it was understood that it exhibited a character that mitigates the nephrotoxic effect of α-CYP. However, both biochemical and histopathological findings suggest that the therapeutic effects of CAPE against α-CYP are limited. As a result of the data obtained from the study, it was considered that α-CYP is toxic to mammals. Significant ultrastructural damage in the renal tubules, glomerular atrophy, and the intensity of inflammatory cell infiltration were found to be correlated with the oral dose of α-CYP. In this study, it was concluded that the misuse or excessive use of pesticides, which are widely used in many fields, and α-CYP in particular may pose a toxic character for non-target organisms.
